# Fe_3_O_4_/granular activated carbon as an efficient three-dimensional electrode to enhance the microbial electrosynthesis of acetate from CO_2_

**DOI:** 10.1039/c9ra06255f

**Published:** 2019-10-23

**Authors:** Hao Zhu, Zhiwei Dong, Qiong Huang, Tian-shun Song, Jingjing Xie

**Affiliations:** State Key Laboratory of Materials-Oriented Chemical Engineering, Nanjing Tech University Nanjing 211816 PR China tshsong@njtech.edu.cn; College of Life Science and Pharmaceutical Engineering, Nanjing Tech University Nanjing 211816 PR China; Jiangsu Collaborative Innovation Center of Atmospheric Environment and Equipment Technology, Jiangsu Key Laboratory of Atmospheric Environment Monitoring and Pollution Control (AEMPC), Nanjing University of Information Science & Technology Nanjing 210044 PR China; Jiangsu National Synergetic Innovation Center for Advanced Materials (SICAM) Nanjing 211816 PR China

## Abstract

Microbial electrosynthesis (MES) allows the transformation of CO_2_ into value-added products by coupling with renewable energy. The enhancement in the microbial activity and electron transfer rate *via* a new electrode modification method is essential for developing MES. Here, three groups of granular activated carbon decorated by Fe_3_O_4_ (Fe_3_O_4_/GAC) with mass fractions of 23%, 38% and 50% were prepared and compared with bare GAC. The volumetric acetate production rate of MES with Fe_3_O_4_/GAC-38% was the highest (0.171 g L^−1^ d^−1^), which was 1.4 times higher that of the control (bare GAC), and the final acetate concentration reached 5.14 g L^−1^ within 30 days. Linear sweep voltammetry and microbial community analyses suggested that Fe_3_O_4_/GAC facilitates extracellular electron transfer and improves the enrichment of electrochemically active bacteria. Fe_3_O_4_/GAC is an effective three-dimensional electrode material that enhances biofilm activity on GAC and improves MES efficiency.

## Abstract 2

Microbial electrosynthesis (MES) allows the transformation of CO_2_ into value-added products by coupling with renewable energy.

## Graphical abstract

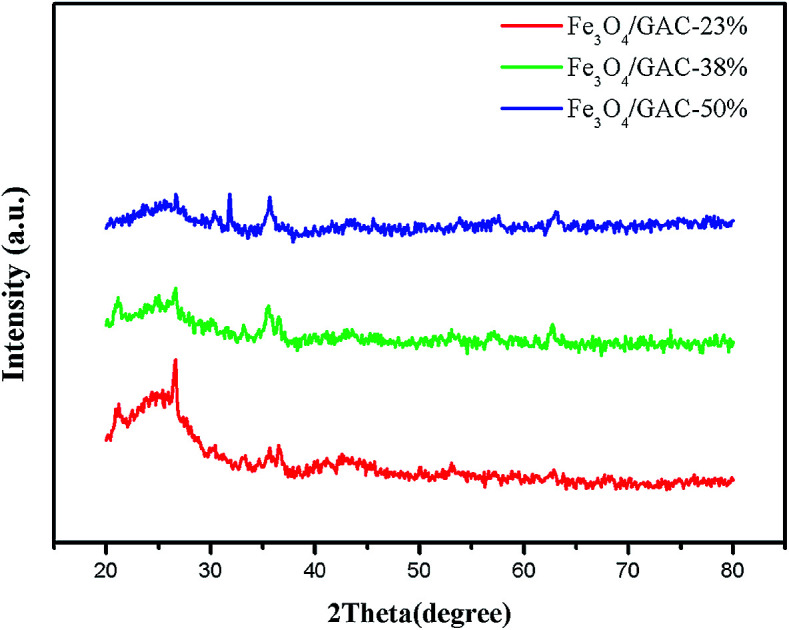

## Introduction

Excessive carbon dioxide (CO_2_) emissions cause serious environmental problems, such as the global rising of temperature.^1^ The use of CO_2_ as feedstock for chemicals has received considerable attention, mainly by chemical transformations, photochemical reactions, electrochemical reductions, and biological conversions.^2^ Compared to those processes that require large areas of land, high energy input or expensive catalysts, microbial electrosynthesis (MES) is a promising approach to transforming CO_2_ into value-added compounds.^3,4^ In MES, electroautotrophic microbes can directly or indirectly utilize electrons *via* H_2_ from a cathode to reduce CO_2_ to multi-carbon organics^5,6^ using biocatalysts with little electrical energy input and high CO_2_ transformation efficiency.

Since the electrons provided by the cathode are the only electron supplies of electroautotrophic microbes, increasing the bacterial extracellular electrical transfer rate (EET) may significantly benefit the MES processing efficiency. Many efforts to enhance the microbe–cathode interactions have been devoted to increasing the biocompatibility porosity,^7^ augmenting the catalytic activity,^8^ incorporating positively charged functional groups,^9^ and improving biofilm conductivity.^10^ In addition, fluidized three-dimensional electrodes, such as granular activated carbon (GAC), have been used to improve the indirect electron transfer rate between planktonic electroautotrophic microbes and the cathode in MES.^11^

Iron(iii) oxide was reported to be a natural terminal electron acceptor that helps to improve EET^12^ with advantages of good biocompatibility, large specific surface area and low toxicity.^13,14^ Magnetite (Fe_3_O_4_), as a bioavailable Fe(iii) oxide, has exhibited a unique electric property due to its electron transfer between Fe(ii) and Fe(iii) in octahedral sites^15^ and high affinity for c-type decaheme cytochromes (OmcA and MtrC) on the outer membranes.^16^ Although bare Fe_3_O_4_ has been utilized for general electrochemical applications,^17,18^ Fe_3_O_4_ particles have the drawback of aggregation due to nanometer-scale and magnetic effects, which can remarkably decrease their catalytic activity.^19^ GAC is commonly used as a carrier for microorganisms and catalysts because it has no biological toxicity, high surface area, and numerous pores that are available for modifications or adsorption.^20^ It has been reported that Fe_3_O_4_ nanoparticles grown on the carbon supports^21,22^ have better catalyst dispersibility to enhance the performance of microbial fuel cells (MFC). Thus, loading Fe_3_O_4_ onto GAC not only reduces the aggregation of the Fe_3_O_4_, but also improves the conductivity of GAC and provides more electron transfer sites. Thus, the coupling of Fe_3_O_4_ with GAC has great potential to further enhance the EET and improve the performance of the MES.

Herein, a three-dimensional electrode, GAC decorated by Fe_3_O_4_ has been constructed by a coprecipitation process to explore its impact on the performance of MES systems. The MES with bare GAC was used as the control, and the effect of the iron oxide content on the acetate production rate in the MES was also investigated.

## Materials and methods

### Preparation of Fe_3_O_4_/GAC

GAC was purchased from Shanghai (China) Activated Carbon Co. Ltd. The specific surface area of GAC and average pore diameter were 900 m^2^ g^−1^ and 2.2 nm, respectively. GAC was washed with deionized water to remove the impurities and was dried in a vacuum oven at 60 °C before use.

Fe_3_O_4_/GAC was prepared by an alkaline co-precipitation process.^23^ FeCl_3_·6H_2_O and FeSO_4_·7H_2_O (molar ration 1 : 2) were dissolved in 400 mL deionized water, followed by the addition of GAC to the solution and stirring for 1 h. The solution was heated to 70 °C under agitation, the pH was adjusted to 10 with 5 mol L^−1^ NaOH solution, the suspension was stirred until cooled, and then washed with deionized water to pH 7. Finally, Fe_3_O_4_/GAC was dried at 105 °C and stored in a desiccator until use. The amount of GAC used during the synthesis was varied to produce Fe_3_O_4_/GAC having mass ratios (w/w, iron oxide/GAC) of 23%, 38% and 50%. In this experiment, bare GAC was used as a control.

### Construction and operation of MES

The MES was constructed using a dual-chamber reactor separated by a proton exchange membrane (Nafion 117, Dupont Co., USA). The anode was a titanium mesh with iridium and ruthenium coating (50 mm × 25 mm × 1 mm, length × width × thickness, Baoji Longsheng Nonferrous Metal Co. Ltd., China). Carbon felt (50 mm × 50 mm × 5 mm, length × width × thickness) was utilized as a cathode in all of the reactors. The anode and cathode were suspended in 280 mL of media in two chambers. In addition, 16 g L^−1^ of Fe_3_O_4_/GAC were added to the cathode chamber of MES, whereas MES with GAC was used as the control group. The cathode potential was set at −1.05 V *vs.* Ag/AgCl reference electrode using a potentiostat (CHI1000C, Shanghai Chenhua Instrument Co. Ltd.). The cathodic compartment was filled with growth medium;^10^ the anodic medium consisted of 50 mL L^−1^ PETC salt solution, 6 g L^−1^ NaCl, and 2 g L^−1^ KCl. Mixed culture from the long-running MES was transferred (5% v/v) to the cathodic chamber under a hydrogen-containing gas mixture (CO_2_ : H_2_; 20 : 80) with mild stirring (150 rpm) to facilitate biofilm growth on the cathode and GAC. After 7 days of experiment under a hydrogen-containing gas mixture, fresh growth medium was used to replace the cathodic medium, then the gas mixture was switched to 100% CO_2_ (3 mL min^−1^) and continually gassed into the cathodic chamber. All the reactions were performed in duplicate and maintained at room temperature (25 ± 2 °C).

### Analytical methods

X-ray diffraction (XRD, Rigaku Smartlab 3 kW) patterns were recorded with Cu Kα radiation (1.54 Å) in the 2*θ* range of 20–80° to identify the structures of Fe_3_O_4_/GAC. The surface morphologies of the cathode surfaces were studied *via* scanning electron microscopy with coupled energy-dispersive spectroscopy (SEM-EDS; JSM-5900, Japan). The current was continuously monitored using a precision multimeter and a data acquisition system (Keithley Instruments 2700, USA). Linear sweep voltammetry (LSV) was used to evaluate the cathode properties *via* potentiostat (CHI660D, Shanghai Chenhua Instrument Co. Ltd.). The cathode, anode, and Ag/AgCl were utilized as the working electrode, counter electrode, and reference electrode, respectively, and their scanning potentials ranged from −200 mV to −1100 mV at a scan rate of 1 mV s^−1^. The current density referred to the projected cathode surface. Volatile fatty acid (VFA) was measured using a high-performance liquid chromatography (HPLC) apparatus (Agilent Technologies 1260, USA). Coulombic efficiency (CE) was calculated as follows: CE = *C*_P_/*C*_T_ × 100%, where *C*_T_ is the total coulomb consumption calculated by integrating the area under the current–time curve (*i*–*t* curve); *C*_P_ is the coulomb consumption in the product, and its formula is *C*_P_ = *b* × *n* × *F*, where *b* is the number of electrons in the product (8 eq mol^−1^); *n* is the number of moles of the product; and *F* is the Faraday constant (96 485 C mol^−1^).

### Iron reduction rate (FER) measurement

FER was measured based on previous literature reports.^24^ The formulation of the nutrient solution consisted of (per liter of de-ionized water): 2.5 g NaHCO_3_, 0.1 g KCl, 0.6 g NaH_2_PO_4_·H_2_O, 1.5 g NH_4_Cl, 2.2 g glucose and 7.3 g ferric citrate. The sterilized nutrient solution was distributed into a 100 mL anaerobic bottle (effective volume 40 mL per bottle). Oxygen was removed by blowing pure N_2_ for 10 minutes to create an anaerobic environment. At the end of the experiment, the Fe_3_O_4_/GAC and bare GAC were taken out and vortexed in distilled water to obtain a bacterial suspension. A 5% (v/v) bacterial suspension was placed in the anaerobic bottle and incubated at 28 °C. Samples were taken out every 12 hours and the ferrous content was analyzed. The phenanthroline method was used to measure the ferrous content.^25^

### Microbial community

Samples from the inoculum, GAC and Fe_3_O_4_/GAC were collected at the end of the experiment. Prior to DNA extraction, the biofilms on GAC and Fe_3_O_4_/GAC were acquired by vortex oscillation. Total genomic DNA was extracted from the samples by using a PowerSoil DNA isolation kit (MO BIO Laboratories Inc., USA) in accordance with the manufacturer's protocol. The DNA quality was assessed at a ratio of 260 nm/280 nm through a NanoDrop spectrophotometer (ND-2000, NanoDrop Technologies Inc., USA), and highly pure genomic DNA (*A*_260_/*A*_280_ = 1.8) was used for Illumina high-throughput sequencing by GENEWIZ (Suzhou, China).

## Results and discussion

### Structural properties

**Fig. 1:**
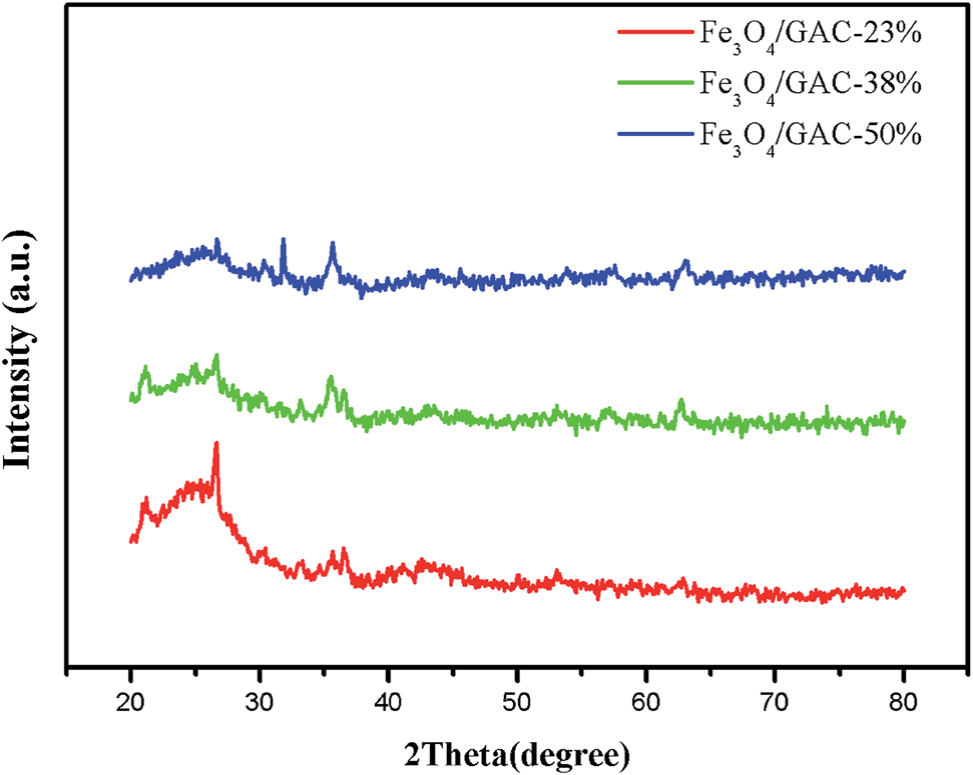
XRD patterns of different Fe_3_O_4_/GAC.

The crystalline structures of prepared Fe_3_O_4_/GAC were evaluated by using XRD (Fig. 1). Fe_3_O_4_/GAC exhibited the characteristic diffraction peaks at 30.2°, 35.5° and 62.8°, which represent the (220), (311) and (440) reflection planes of the cubic inverse spinel structure of Fe_3_O_4_ nanoparticles, respectively,^26^ while the peak at 26.34° was related to graphite-like carbon.^27^ It was also found that as the Fe_3_O_4_ loading increased, the carbon characteristic peak decreased continuously.

**Fig. 2:**
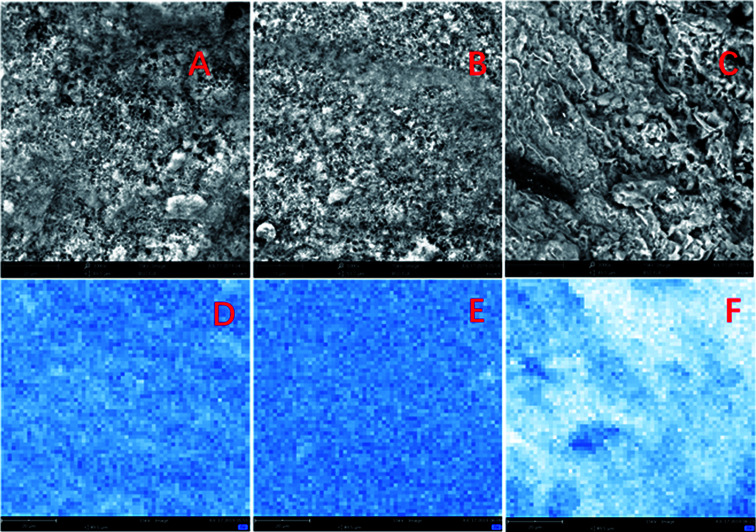
SEM images of (A) Fe_3_O_4_/GAC-23%, (B) Fe_3_O_4_/GAC-38%, (C) Fe_3_O_4_/GAC-50% and the elemental distribution on the iron map of (D) Fe_3_O_4_/GAC-23%, (E) Fe_3_O_4_/GAC-38% and (F) Fe_3_O_4_/GAC-50%.

The morphological properties of prepared Fe_3_O_4_/GAC were evaluated by using SEM (Fig. 2). According to the SEM image (Fig. 2A), Fe_3_O_4_ nanoparticles were effectively anchored and uniformly distributed over the GAC. The spherical configuration and smooth contours of Fe_3_O_4_ nanoparticles were identically maintained on the GAC supports. As the Fe_3_O_4_ concentration increased, more and more small particles were loaded on the GAC. The particles were densely and uniformly distributed in Fe_3_O_4_/GAC-38% (Fig. 2B). However, as the Fe_3_O_4_ loading increased to 50%, the distribution of the particles was no longer uniform, but some clumps were formed (Fig. 2C). This phenomenon indicated that Fe_3_O_4_ particles had been agglomerated on GAC. Furthermore, the iron loading was evaluated by mapping the iron distribution on GAC. The iron was evenly distributed on Fe_3_O_4_/GAC_4_-23% (Fig. 2D) and Fe_3_O_4_/GAC-38% (Fig. 2E), but when the Fe_3_O_4_ loading increased to 50%, the distribution of the iron was obviously non-uniform (Fig. 2F). This phenomenon may be due to the agglomeration of Fe_3_O_4_ on the surface of the GAC, which is consistent with the SEM observation.

### Microbial electrosynthesis of acetate from CO_2_

**Fig. 3:**
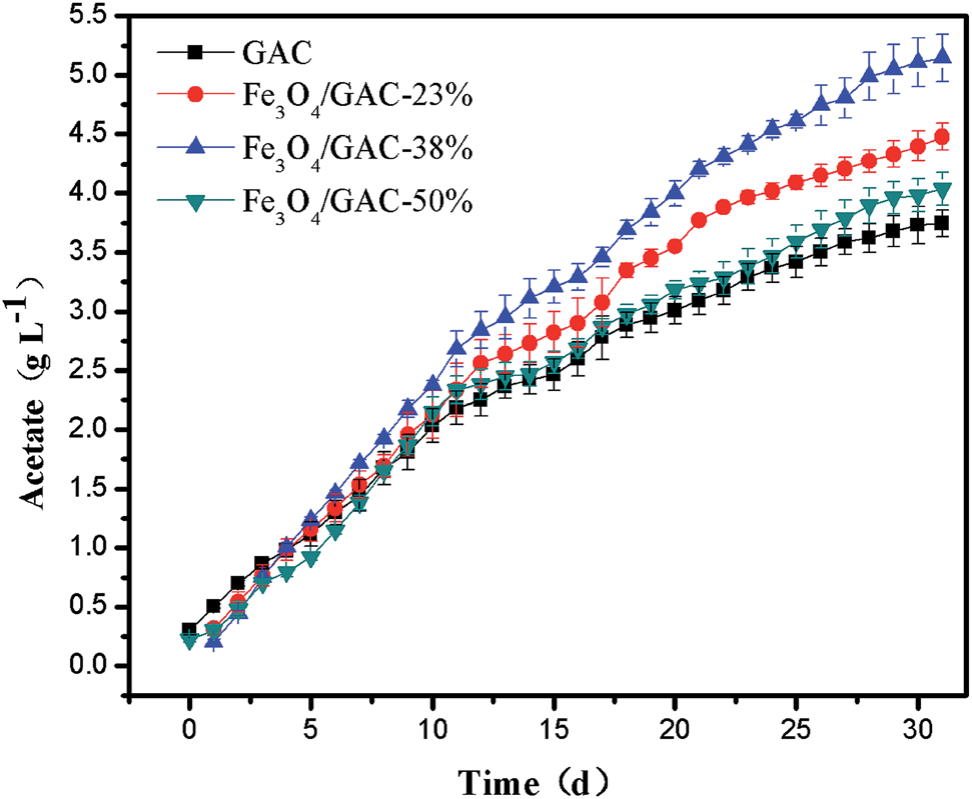
Acetate production over time with different Fe_3_O_4_/GAC.

Acetate was the predominant product formed immediately in both reactors (Fig. 3). The acetate concentration continuously increased with time in all of the MES systems. On day 30, the acetate concentration produced in MES with Fe_3_O_4_/GAC-38% was the highest (5.14 ± 0.2 g L^−1^), followed by MES with Fe_3_O_4_/GAC-23% (4.47 ± 0.11 g L^−1^) and Fe_3_O_4_/GAC-50% (4.04 ± 0.13 g L^−1^), while the acetate concentration of control (3.74 ± 0.16 g L^−1^) was the lowest.

The volumetric acetate production rate of the control was 0.123 g L^−1^ d^−1^ for 30 days of reaction (Table 1). As the iron oxide content increased, the volumetric acetate production rate increased, the volumetric acetate productivity in MES with Fe_3_O_4_/GAC-23% and Fe_3_O_4_/GAC-38% was 0.149 g L^−1^ d^−1^ and 0.171 g L^−1^ d^−1^, respectively, which increased by nearly 1.2–1.4 times as compared with the control. However, when the Fe_3_O_4_ loading increased to 50%, the volumetric acetate productivity decreased to 0.134 g L^−1^ d^−1^. This might be due to the agglomeration of Fe_3_O_4_ on the GAC, which would decrease the catalytic activity of Fe_3_O_4_ and cause the low acetate production rate in MES.

**Table 1:** Acetate production in MES with different Fe_3_O_4_/GAC

	Volumetric production rate (g L^−1^ d^−1^)	Surface based rate (g m^−2^ cathode d^−1^)	Current (mA)	Max. acetate titer (g L^−1^)	Coulombic efficiency (%)
GAC	0.123	13.9	8.3	3.74	55.1
Fe_3_O_4_/GAC-23%	0.149	16.7	11.4	4.47	63.0
Fe_3_O_4_/GAC-38%	0.171	19.2	12.6	5.14	73.1
Fe_3_O_4_/GAC-50%	0.134	15.1	11.7	4.04	49.9

**Fig. 4:**
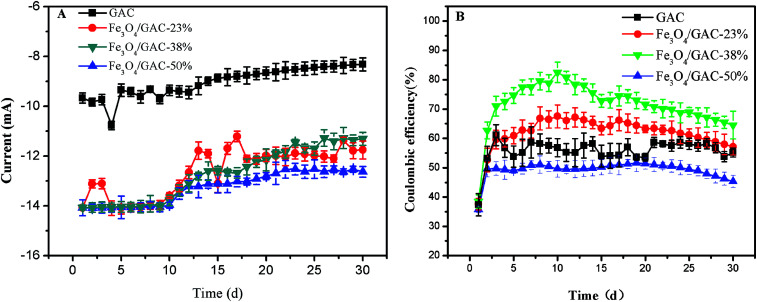
Current (A) and CE (B) over time with different Fe_3_O_4_/GAC.

The current was generated (Fig. 4A) immediately after inoculation and decreased gradually. At the end of the experiment, the current of the control was only 8.30 ± 0.26 mA, which was lower than that of the MES with Fe_3_O_4_/GAC. The highest current was observed in MES with Fe_3_O_4_/GAC-38% (12.6 ± 0.21 mA). The CE was also calculated (Fig. 4B). The CE of the control remained at approximately 55.1%, whereas that of the MES with Fe_3_O_4_/GAC was maintained at 49.9–73.1%. The CE of MES with Fe_3_O_4_/GAC-50% was lower than that of the control, and the MES with Fe_3_O_4_/GAC-38% had the highest CE, followed by Fe_3_O_4_/GAC-23%. The results implied that Fe_3_O_4_/GAC-38% was most beneficial for the recycling of electrons and product generation.

### Bioelectrocatalytic activity

**Fig. 5:**
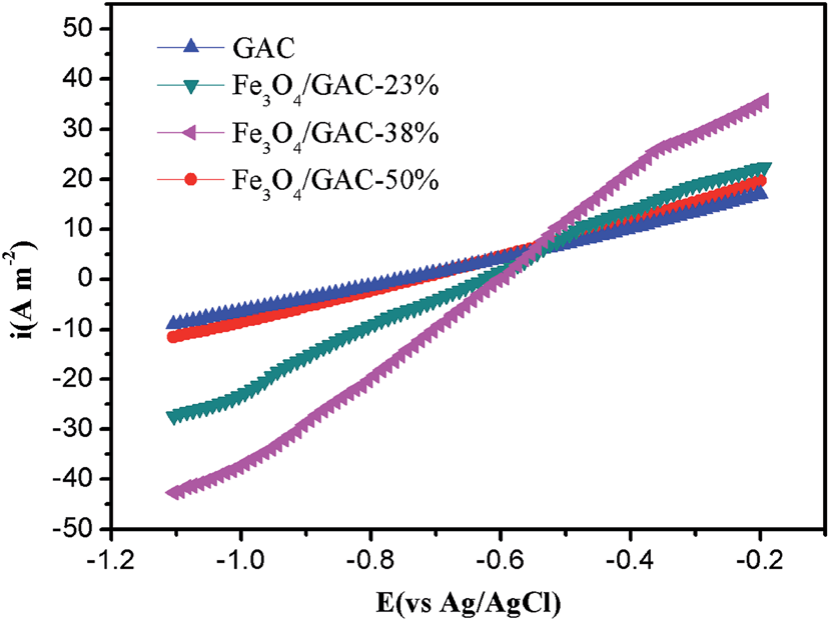
LSV on MES with different Fe_3_O_4_/GAC.

LSV was used to understand the effect of the different cathodes on the catalytic activities. The cathodic current density increased as the iron loading increased. The highest cathodic current density of MES with Fe_3_O_4_/GAC-38% was 40.3 A m^−2^ at −1.05 V (Fig. 5), but when the iron loading further increased to 50%, the cathodic current densities decreased to 10 A m^−2^, which was lower than that of Fe_3_O_4_/GAC-23% (28.4 A m^−2^). The control had the lowest cathodic current density of 7.7 A m^−2^. The cathodic current density of MES with Fe_3_O_4_/GAC-38% was 5.2 times higher than that of the control. The LSV results implied that Fe_3_O_4_/GAC-38% had the highest electrocatalytic activity.

### Iron reduction rate

**Fig. 6:**
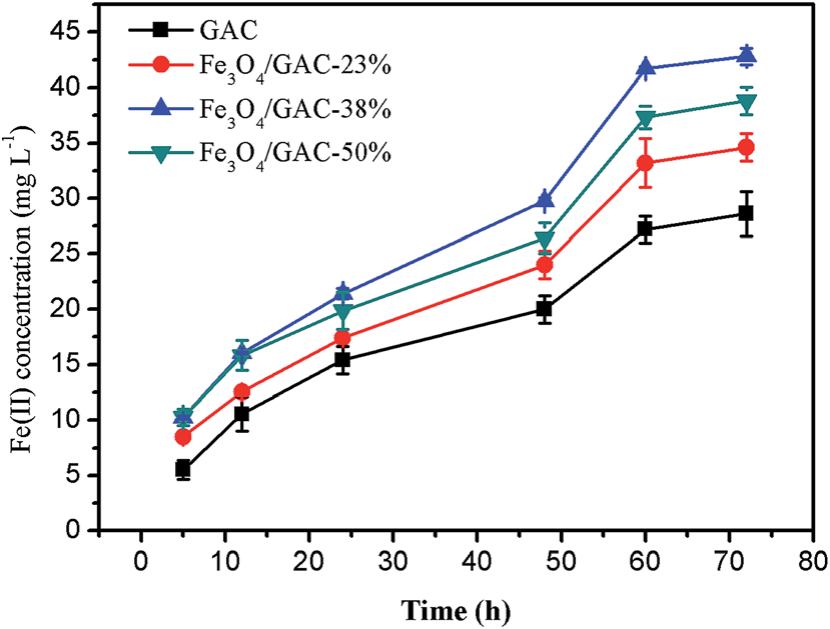
Concentration of Fe(ii) reduced by bacteria on different Fe_3_O_4_/GAC.

During the first 24 hours of incubation, the concentrations of Fe(ii) in all groups accumulated slowly (Fig. 6). The concentration of Fe(ii) began to increase quickly after 24 h, probably due to the microbial growth and enrichment in the first 24 h. The Fe(ii) reduction rate in the control was the lowest (0.39 ± 0.02 mg L^−1^ h^−1^). The Fe(ii) reduction rate of Fe_3_O_4_/GAC-38% was the highest (0.58 ± 0.01 mg L^−1^ h^−1^), followed by Fe_3_O_4_/GAC-50% (0.55 ± 0.02 mg L^−1^ h^−1^) and Fe_3_O_4_/GAC-23% (0.52 ± 0.01 mg L^−1^ h^−1^). The iron reduction rate represents the activity of iron-reducing bacteria. Most of the iron-reducing bacteria are exoelectrogens,^28^ which are capable of exchanging electrons with the solid surface. The high iron reduction rate in Fe_3_O_4_/GAC-38% implied that its activity toward iron-reducing bacteria was enhanced and the electron transfer rate was improved. This might be one of the reasons why the highest volumetric acetate production rate was obtained by Fe_3_O_4_/GAC-38%.

### Microbial community

**Fig. 7:**
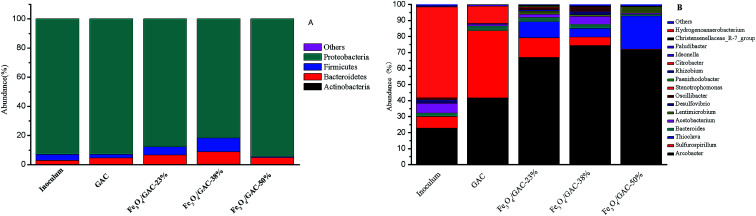
Relative abundance of bacterial phylum communities (A), genus communities (B) in inoculum, GAC and Fe_3_O_4_/GAC.

The microbial communities in the inoculum, biofilms on the GAC and Fe_3_O_4_/GAC were analyzed at the end of the experiments. Proteobacteria was the most abundant phylum in the microbial communities in all of the groups (Fig. 7A); it accounted for 93% in the inoculum and the change in its abundance was small in other groups except Fe_3_O_4_/GAC-38%, which had a relatively low abundance (81.7%). The other two most abundant phyla were Bacteroidetes and Firmicutes.

The microbial communities were also identified at a genus level (Fig. 7B). The main genus in the microbial community of the inoculum was *Citrobacter* (56.8%), followed by *Arcobacter* (22.7%) and *Sulfurospirillum* (7.2%). *Citrobacter* has been reported to be a biohydrogen-production bacterium,^29^ which might utilize organic matter to produce hydrogen. The *Arcobacter* genus has been reported to be an electrochemically active bacterium that can transfer electrons with the electrode. *Sulfurospirillum* may switch between CO_2_ fixation and acetate oxidation based on the context and environment.^30^*Acetobacterium* was the 4^th^ most abundant group (6.0%) in the inoculum, which can convert CO_2_ into acetate by directly using an electrode or H_2_ as an electron donor.^31,32^

When the inoculum was added to MES, the abundance of the microbial communities changed after 30 days of reaction. In MES-GAC, *Sulfurospirillum* became the most abundant genus, and it is reported to be microaerophilic.^30^ It maintained an anaerobic environment for the rest of the community by expanding the trace amounts of oxygen that diffused from the anode. Another group with relatively high abundance was *Arcobacter* (41.5%). The enrichment of such microorganisms may be stimulated from the electrochemical environment and increased interspecies electron transfer by carbon particles.^33^ When Fe_3_O_4_/GAC was added to the MES, the abundance of *Arcobacter* dramatically increased. Among all the groups, the abundance of the *Arcobacter* in MES with Fe_3_O_4_/GAC-38% was the highest (74.3%), followed by Fe_3_O_4_/GAC-50% (71.5%) and Fe_3_O_4_/GAC-23% (66.8%). This may be related to the stimulation of the activity of iron reduction bacteria by ferric irons, which is conducive to electron transfer; this result is consistent with the iron reduction rate. Compared with Fe_3_O_4_/GAC-23%, the abundance of *Arcobacter* and *Acetobacterium* increased in Fe_3_O_4_/GAC-38%, thus leading to the highest acetate production rate. Although in Fe_3_O_4_/GAC-50%, the abundance of *Arcobacter* increased, the abundance of *Acetobacterium* significantly decreased. This might be due to the high dosage of Fe_3_O_4_ causing aggregation (Fig. 2), reducing the electron transfer rate and microbial enrichment, and thus decreasing the acetate productivity.

### Practical significance and perspectives

In the MES process, electroautotrophic bacteria uptake electrons from the cathode to produce value-added chemicals using CO_2_ as the sole carbon source; therefore, the electron transfer rate between the cathode and bacteria is an important constraining step for MES performance improvement. Many strategies have been devoted to improving the electron transfer rate, such as reticulated vitreous carbon (RVC) with large specific surface area,^7,34^ carbon cloth with bioinorganic networks of rGO-TEPA,^35^ rGO/biofilm with high electronic conductivity,^10^ carbon cloth with positively charged polyaniline^9^ and metal framework of nickel foam with graphene.^36^ The highest acetate production rate based on the surface area of the cathode in MES with RVC^34^ was 25.2 g m^−2^ d^−1^ with CE of 100% but the volumetric acetate production rate in MES with RVC was not the highest. This is mainly due to the low cathode volume in relation to the catholyte volume. LaBelle *et al.*^37^ reported the highest volumetric acetate production rate (3.1 g L^−1^ d^−1^) but the packing density of graphite granules was too high (500 g L^−1^), which actually limited the cathodic solution volume. Electron transfer between graphite granules and a current collector is unfavorable on account of the irregular porosity and shape of graphite granules and biomass blockage. In our previous report,^11^ a fluidized GAC (16 g L^−1^) promoted the growth of biofilm on GAC and reduced resistance due to biomass clog; thus, the electron transfer in MES with the fluidized GAC electrode was more efficient. More importantly, the role of biofilm on the three-dimensional electrode (GAC) might be enhanced, while the usual strategy was used to improve the microbial activity of biofilm on the cathode. In the present study, Fe_3_O_4_/GAC was prepared by an alkaline co-precipitation process to further improve the role of biofilm on GAC. A relatively high volumetric acetate production rate (0.17 g L^−1^ d^−1^) was obtained in MES with Fe_3_O_4_/GAC. H_2_ can be produced electrochemically *via* water electrolysis at the cathode potential of −1.05 V *vs.* Ag/AgCl. GAC was beneficial to the biofilm formation by the indirect extracellular electron transfer *via* H_2_ from the cathode and enhanced the efficiency of CO_2_ reduction in MES. Furthermore, Fe_3_O_4_ was favorable for electroautotrophic microbes on GAC to accept electrons, thus Fe_3_O_4_/GAC can facilitate extracellular electron transfer and improve the enrichment of electroautotrophic microbes. If scalable, this fluidized Fe_3_O_4_/GAC will provide a large surface area for microbial colonization in solution and the mass transfer effect will be further improved by introducing some stirring or gas sparging mixing equipment on a large scale. The acetate production rate may be enhanced as the activity of the biofilm on GAC increases. Furthermore, Fe_3_O_4_/GAC^38^ has magnetic properties, which allow recycling in the reactor. Compared to bare GAC, the magnetic separability would also allow its application in product separation in MES^39,40^ and improve the biosynthesis efficiency of acetate.

## Conclusion

Fe_3_O_4_ nanoparticles anchored over GAC were prepared through an alkaline coprecipitation process and used as a three-dimensional electrode in the MES system. The decoration of Fe_3_O_4_ on the GAC improved the EET efficiency and the relative abundance of *Arcobacter* was higher in the presence of Fe_3_O_4_/GAC. The volumetric acetate production of MES with Fe_3_O_4_/GAC-38% increased by 1.4 times as compared with that of the control (bare GAC), which suggests that the Fe_3_O_4_/GAC composite could be an effective three-dimensional electrode material to improve MES efficiency.

## Conflicts of interest

There are no conflicts to declare.

## Supplementary Material
